# Monoclonal anti-endoglin antibody TRC105 (carotuximab) prevents hypercholesterolemia and hyperglycemia-induced endothelial dysfunction in human aortic endothelial cells

**DOI:** 10.3389/fmed.2022.845918

**Published:** 2022-09-07

**Authors:** Katarina Tripska, Ivone Cristina Igreja Sá, Martina Vasinova, Matej Vicen, Radim Havelek, Samira Eissazadeh, Zuzana Svobodova, Barbora Vitverova, Charles Theuer, Carmelo Bernabeu, Petr Nachtigal

**Affiliations:** ^1^Department of Biological and Medical Sciences, Faculty of Pharmacy in Hradec Kralove, Charles University, Hradec Kralove, Czechia; ^2^Department of Medical Biochemistry, Faculty of Medicine in Hradec Kralove, Charles University, Hradec Kralove, Czechia; ^3^Tracon Pharmaceuticals, Inc., San Diego, CA, United States; ^4^Centro de Investigaciones Biológicas Margarita Salas, Consejo Superior de Investigaciones Científicas, Madrid, Spain

**Keywords:** endoglin, TRC105, 7-ketocholesterol, high glucose, endothelial dysfunction

## Abstract

Endoglin (Eng) is a co-receptor of the transforming growth factor β superfamily playing an important role in endothelial dysfunction. TRC105 (carotuximab) is a monoclonal antibody that blocks Eng and its downstream Smad signaling pathway. Here we have investigated for the first time the effects of TRC105 treatment on the development of endothelial dysfunction induced by 7-ketocholesterol (7K) or high glucose (HG), focusing on Eng expression, signaling, and function. In the hypercholesterolemia study, human aortic endothelial cells (HAoECs) were treated with TRC105 (300 μg/ml) for 1 h, followed by the addition of 7K (10 μg/ml) for another 12 h. In the hyperglycemia study, HAoECs were exposed to HG (45 mM) for 60 h, followed by the addition of TRC105 for another 12 h, and cells treated with 5mM glucose and 40 mM mannitol served as control. Protein levels, adhesion, and transmigration of monocytes were assessed by flow cytometry, mRNA expression was measured by qRT-PCR. 7K and HG treatment increased protein levels of NF-κB and Eng and adhesion and transmigration of monocytes through HAoECs monolayer. TRC105 pretreatment reduced the 7K- or HG-induced Eng protein levels and pSmad1/5 and pSmad2/3 signaling. Despite increased protein levels of P-selectin and VCAM-1, TRC105 mediated blockage of Eng prevented 7K- and HG-induced adhesion and transmigration of monocytes through endothelial monolayers. These results suggest that TRC105-mediated Eng blockage can counteract the hypercholesterolemia- and hyperglycemia-induced endothelial dysfunction in HAoECs, suggesting that Eng might be a potential therapeutic target in disorders associated with elevated cholesterol and glucose levels.

## Introduction

Hyperglycemia and hypercholesterolemia are risk factors for the development of endothelial dysfunction and are hallmarks of the so-called metabolic syndrome. Metabolic syndrome represents a cluster of conditions that co-occur and are considered the risk factors for cardiometabolic disorders such as atherosclerosis, ischemic heart disease, or type II diabetes mellitus (1). Cholesterol in the blood, particularly that deposited in the artery intima, can be altered by several processes, culminating in the production of oxysterols that can induce endothelial dysfunction (2, 3). LDL is mostly composed of non-enzymatic oxysterols and among these, 7-ketocholesterol (7K) is a typical representative (4), and it is also one of the most frequent oxysterols found in healthy human plasma (5).

Endoglin is a glycoprotein that is a co-receptor for members of the transforming growth factor β (TGF-β) superfamily (6). There are two different forms of endoglin: (i) full-length membrane-bound endoglin (Eng), and (ii) soluble endoglin (sEng). The membrane form is expressed predominantly in endothelial cells (7) but also in vascular smooth muscle cells (8), fibroblasts (9), hepatic stellate cells (10), and activated monocytes and macrophages (11). sEng is the cleavage product of the extracellular domain of Eng upon the enzymatic activity of matrix metalloproteinases (MMP-14 and MMP-12) (12, 13). It is released into the circulation in various pathological conditions, such as arterial hypertension, type II diabetes mellitus (14), non-alcoholic steatohepatitis (15), familial hypercholesterolemia (16, 17), and preeclampsia (18); and also in patients with atherosclerosis who had increased levels of total cholesterol, LDL, triglycerides and decreased levels of HDL (19). Moreover, increased levels of sEng correlate with complications of diabetes, such as retinopathy, peripheral neuropathy, or nephropathy (20–23). Although there is limited information in the literature regarding the levels of MMP-12 in the above-mentioned conditions, it should be noted that MMP-14 has been shown to be up-regulated in the human fetoplacental endothelium during gestational diabetes mellitus (24). Levels of sEng and MMP-14 were found to be increased simultaneously in both hyperglycemia and hypoglycemia models and this effect could be reversed with the anti-diabetic drug glucagon-like peptide-1 (GLP-1) (25). In addition, levels of MMP-14 were significantly decreased after 6 months of therapy with pitavastatin, a drug used to decrease cholesterol levels (26).

In endothelial cells, Eng associates with transforming growth factor-β (TGF-β) receptors type I (ALK-1, ALK-5) and type II (TGF-βRII, BMPRII, ActR2A, ActR2B) and enables the binding of ligands of the TGF-β family, including bone morphogenetic proteins 9 and 10 (BMP9 and BMP10) (27, 28). This receptor complex can trigger intracellular signaling mediated by Smad proteins, including the ALK-1/pSmad1/5 or ALK-5/pSmad2/3 signaling pathway (29). Furthermore, Eng is a mediator of the delicate balance between ALK-1/pSmad1/5 and ALK-5/pSmad2/3 signaling and regulates whether endothelial cells are activated or quiescent (30, 31).

*In vivo* studies have shown that Eng is critically involved in the pathophysiological functions of the endothelium in the cardiovascular system, including the prevention of endothelial dysfunction development, the initial step in atherogenesis (1). In this regard, several *in vivo* and *in vitro* studies have suggested different roles of Eng with respect to endothelial dysfunction (1). Rossi et al. demonstrated that reduced Eng expression under inflammatory conditions results in decreased adhesion and transendothelial migration of leukocytes, suggesting that Eng is a cell adhesion molecule (32). The treatment of aortic endothelial cells with the oxysterol 7-ketocholesterol (simulating the effects of oxidized LDL) resulted in induced Eng expression and consequent increase in adhesion and transmigration of monocytes via endothelial monolayer, pointing out to a crucial role of Eng in 7-ketocholesterol-induced leukocyte transmigration and endothelial dysfunction (33). Hyperglycemia represents a hallmark of diabetes mellitus and is involved in the development of endothelial dysfunction as well (34). Of note, Eng expression is upregulated under high glucose conditions (35), but the precise link between hyperglycemia-induced endothelial dysfunction and Eng expression, signaling, and function is not known. Different stimuli related to endothelial dysfunction, such as vascular stress, oxysterols, and inflammation, have been reported to upregulate Eng gene transcription via transcription factors Kruppel Like Factor 6 (KLF6), liver X receptor alpha (NR1H3) and NF-κB p65 (RELA) (33, 36–38).

Taken together, the above data point out to Eng as an interesting therapeutical target for the modulation of endothelial dysfunction development in diseases with elevated cholesterol and glucose levels. TRC105 (carotuximab), a monoclonal antibody that binds to Eng and modulates Smad signaling, was initially developed to be used in oncology by targeting proliferating endothelium in the vasculature of solid tumors (39, 40). TRC105 has also been studied in clinical trials to treat the abnormal angiogenesis of acute macular degeneration (39). However, in the context of endothelial dysfunction development and associated risk factors, no studies are available on the potential role of TRC105 treatment on Eng expression, signaling, and function. Therefore, in this study, we hypothesized that TRC105 treatment could prevent the 7-ketocholesterol- and hyperglycemia-induced development of endothelial dysfunction by a direct effect on Eng expression, signaling, and function.

## Materials and methods

### Cell culture

Primary human aortic endothelial cells (HAoECs), purchased from PromoCell (Heidelberg, Germany), were cultured in 100 mm tissue culture dishes (TPP, Trasadingen, Switzerland) previously coated with gelatin (MilliporeSigma, Burlington, Massachusetts, United States) and grown in EGM-2 medium (Lonza, Basel, Switzerland) with corresponding supplements (Lonza) and 10% fetal bovine serum (FBS; Biosera, Nuaillé, France) at 37°C and 5% CO_2_. Cells were passaged after reaching 70-90% confluence, and all experiments were performed with HAoECs passage 5 (cumulative population doubling 10-11).

The human acute monocytic leukemia cell line (THP-1) was generously provided by Soòa Èejková (Institute for Clinical and Experimental Medicine, Prague, Czechia). Cells were cultured in non-adhesive cell culture flasks (SPL Life Sciences, Gyeonggi-do, South Korea) using RPMI 1640 medium (Thermo Fisher Scientific, Waltham, MA, United States) supplemented with 10% FBS, 1% penicillin-streptomycin (MilliporeSigma), and 2.5% glutamine (Thermo Fisher Scientific). THP-1 cells were passaged when reaching density 8 × 10^5^ cells/ml, and all experiments were conducted with cells up to passage 25.

### Experimental design

**FIGURE 1:**
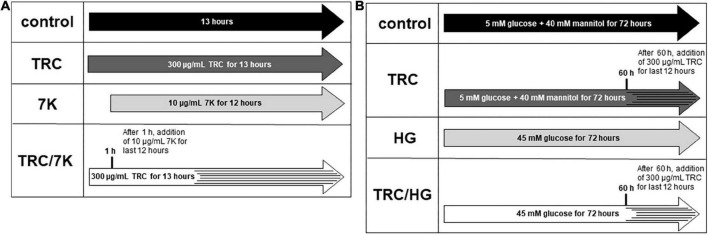
Experimental design. **(A)** Experiments performed with 7-ketocholesterol (7K). **(B)** Experiments performed with high glucose (HG). 7K, 7-ketocholesterol; TRC, TRC105; HG, high glucose. To analyze Smad signaling in HAoECs, cells were serum-starved in EBM-2 medium for 22 h and then pretreated with TRC105 for 90 min. HAoECs were stimulated with either 7-ketocholesterol or glucose for 30 min after TRC105 pretreatment.

TRC105 (TRACON Pharmaceuticals Inc.) was used at a final concentration of 300 μg/ml, reflecting plasma levels in patients treated with TRC105 in phase II clinical trials (41). To study the TRC105 effects in cholesterol-induced endothelial dysfunction, 10 μg/ml 7-ketocholesterol (7K) (MilliporeSigma) was used to simulate the effects of oxidized LDL, as previously described by Vicen et al. (33). HAoECs were treated with or without TRC105 for 1 h, followed by the addition of 7K for another 12 h (Figure 1A). To evaluate TRC105 effect in hyperglycemic conditions, D-glucose (MilliporeSigma) was used at a final concentration of 45 mM (high glucose, HG), dosage selected according to our preliminary results and a previously published paper (42). To compensate for osmotic effects, control cells were incubated with 5 mM D-glucose and 40 mM mannitol (MilliporeSigma). HAoECs were then treated with or without glucose for 60 h, followed by the addition of TRC105 for another 12 h (Figure 1B). All experiments except Smad signaling were performed with a medium containing 10% FBS.

### Real-time RT-qPCR

For isolation of total RNA, TRI Reagent™ Solution (Thermo Fisher Scientific) was used. Afterward, mRNA was converted to cDNA using a High-Capacity cDNA Reverse Transcription Kit (Thermo Fisher Scientific). TaqMan™ Gene Expression Master Mix (Thermo Fisher Scientific) and TaqMan™ Gene Expression Assays, with the following Assay IDs: *Eng* (Hs00923996_m1), *KLF6* (Hs00810569_m1), *RELA* (Hs00153294_m1) and *NR1H3* (Hs00172885_m1) (all from Thermo Fisher Scientific) were used for evaluation of mRNA expression by real-time RT-qPCR with QuantStudio 6 Flex Real-Time PCR software (Thermo Fisher Scientific). The time-temperature profile was as follows: Hold stage: 50°C for 2 min; 95°C for 10 min; PCR stage: 95°C for 15 s, 60°C for 1 min. The relative expression ratio was calculated as previously described (43) and then recalculated to% of control (control being 100%). For the normalization of the data, *GAPDH* (Hs02758991_g1) or *HPRT1* (Hs02800695_m1) were used for hypercholesterolemic or hyperglycemic experiments, respectively.

### Immunofluorescence flow cytometry

HAoECs were rinsed with PBS (MilliporeSigma) and detached from tissue culture dishes using StemPro™ Accutase™ Cell Dissociation Reagent (Thermo Fisher Scientific). Indirect flow cytometry was used to measure the surface expression of Eng, VCAM-1, and P-selectin. Cells were blocked with goat serum (Vector Laboratories Inc., CA, United States), followed by incubation with the mouse monoclonal antibody P4A4 against human Eng (DSHB, Iowa, US; hybridoma contributors: Drs. Wayner and Vercellotti), VCAM-1 (853.823.020) and P-selectin (855.103.020) (both from Diaclone, Besançon Cedex, France) for 1 h at 4°C. Then, cells were rinsed with PBS and incubated with secondary fluorescent-labeled goat anti-mouse antibody Alexa Fluor 488 (Thermo Fisher Scientific, A11001) for 30 min at 4°C. Intracellular flow cytometry was used for the detection of NF-κB p65, pSmad1/5, and pSmad2/3. HAoECs were fixed with 2% paraformaldehyde and permeabilized with 90% methanol. Next, cells were blocked with chicken serum and incubated with primary rabbit monoclonal antibodies against NF-κB p65 (8242T), pSmad1/5 (9516S) or pSmad2/3 (8828S) (all from Cell Signaling Technology, Danvers, MA, United States). Afterward, HAoECs were incubated with secondary fluorescent-labeled chicken anti-rabbit antibody Alexa Fluor 488 (Thermo Fisher Scientific, A21441).

The protein expression was evaluated with a Cytoflex LX Flow cytometer (Beckman Coulter, Brea, CA, United States) and processed by CytExpert Acquisition and Analysis Software, version 2.3 (Beckman Coulter). For every sample, at least 10,000 events were collected and analyzed. The relative expression index was calculated as previously described (44) and then recalculated to% of control (control being 100%).

### Cell adhesion assays

Cell adhesion assays were performed as previously described (33). Briefly, after premedication, THP-1 cells were added to HAoECs monolayers for 1 h. After co-incubation, dishes were rinsed with PBS, and non-adherent cells were removed. HAoECs with adherent THP-1 cells were detached with StemPro™ Accutase™ Cell Dissociation Reagent and the cell mixture was stained for Eng as described above and analyzed with a flow cytometer. Negative cell sorting method in combination with side scatter and forward scatter was used for the detection and count of adherent THP-1 cells.

### Cell transmigration assays

HAoECs were loaded on polycarbonate membranes of cell culture inserts with 3 μm pore size in 6-well plates (Corning, New York, NY, United States) and incubated until 100% confluency. To study the effects of TRC105 in 7K-induced endothelial dysfunction, normal media (control, TRC) and media with 7K (7K, TRC/7K) were added to the bottom compartment. Approximately 100,000 of actively proliferating THP-1 cells, with (TRC, TRC/7K) or without (control, 7K) 300 μg/ml of TRC105, were added into the upper compartment. To study the effects of TRC105 in hyperglycemia-induced endothelial dysfunction, media with 5 mM glucose and 40 mM mannitol (control, TRC) and media with 45 mM glucose (HG, TRC/HG) were added to the bottom compartment. Around 100,000 of actively proliferating THP-1 cells, with (TRC, TRC/HG) or without (control, HG) 300 μg/ml of TRC105 were added into the upper compartment. After 12 h of incubation, transmigrated THP-1 cells from bottom compartments were counted in 10 μL of media by flow cytometry.

### Statistical analysis

The statistical analysis was performed using GraphPad Prism 9.2 software (GraphPad Software Inc., San Diego, CA, United States). The data are presented as mean ± S.E.M. Comparisons between control groups, TRC groups and 7K or HG groups, were carried out using the Kruskal–Wallis test followed by Dunn’s multiple comparisons test. Direct comparisons between 7K *vs* TRC/7K or between HG *vs* TRC/HG were carried out using the Mann–Whitney test. *P*-values <0.05 were considered statistically significant.

## Results

### TRC105 reduces Eng protein levels but does not affect 7K-induced Eng mRNA expression

**FIGURE 2:**
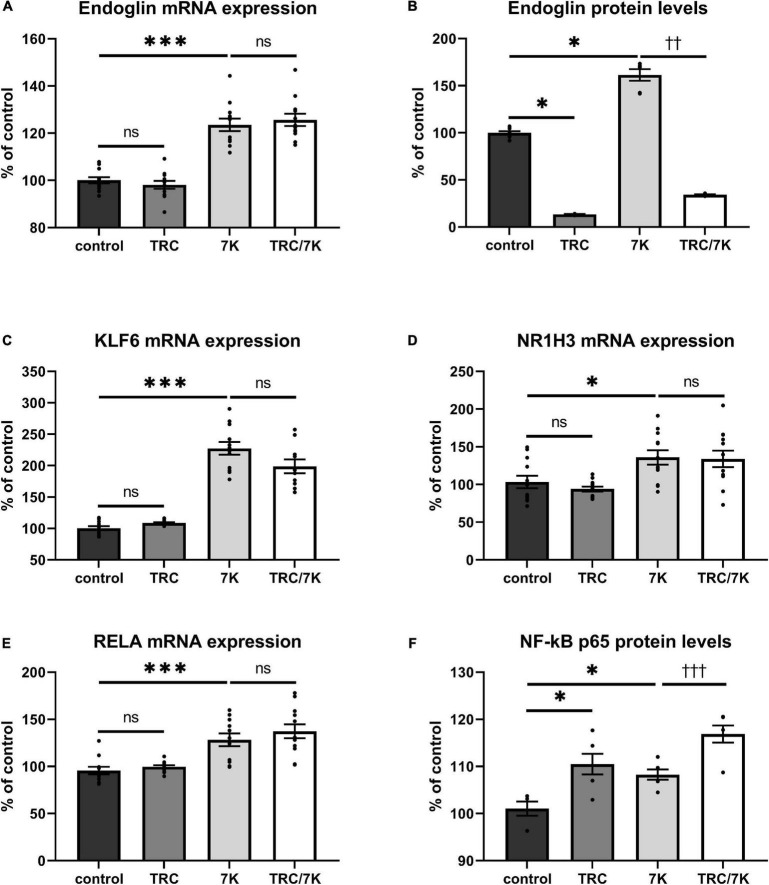
Effect of TRC105 on 7K-induced mRNA and protein expression of Eng, and on the transcription factors KLF6, NR1H3 and RELA (NF-κB p 65) in HAoECs. **(A)** Eng mRNA expression. **(B)** Eng protein levels. **(C)** KLF6 mRNA expression. **(D)** NR1H3 mRNA expression. **(E)** RELA mRNA expression. **(F)** NF-κB p65 protein levels. For the detailed experimental design, please see Figure 1. The data are presented as mean ± S.E.M (*n* = 6). ns, *p* ≥ 0.05; ^∗^*p* < 0.05; ^∗∗∗^*p* < 0.001, using Kruskal–Wallis test for comparisons between control, TRC and 7K, and ^††^*p* < 0.01; ^†††^*p* < 0.001, using Mann–Whitney test for comparison between 7K *vs* TRC/7K.

To evaluate whether TRC105 modulates 7K-induced Eng expression by other mechanisms than direct blockage of the extracellular part of Eng, we measured mRNA expression of Eng and its transcription factors *KLF6*, *RELA*, and *NR1H3* (33). As previously described (33), mRNA and/or protein levels of Eng, KLF6, RELA, and NR1H3 were enhanced by a single treatment with 7K (Figure 2). When analyzing the specific effect of TRC105, no significant change in Eng mRNA expression was found in cells treated with 7K and TRC105 (TRC/7K) when compared to cells treated only with 7K (Figure 2A). However, Eng protein levels were significantly decreased after treatment with TRC105 (with or without 7K) (Figure 2B).

In agreement with the unaffected Eng mRNA expression in the presence of TRC105, there was no significant change in the mRNA expression levels of Eng gene transcription factors KLF6, NR1H3, and RELA (Figures 2C–E). Nevertheless, we observed a slight increase in NF-κB p65 (protein encoded by *RELA*) protein expression after administration of 7K plus TRC105 compared to cells treated only with 7K (Figure 2F).

These data suggest that upon binding to Eng, TRC105 in the presence of 7K, does not affect the levels of Eng mRNA but reduces Eng protein levels, likely via a post-translational modification.

### TRC105 prevents 7K-induced Smad signaling

**FIGURE 3:**
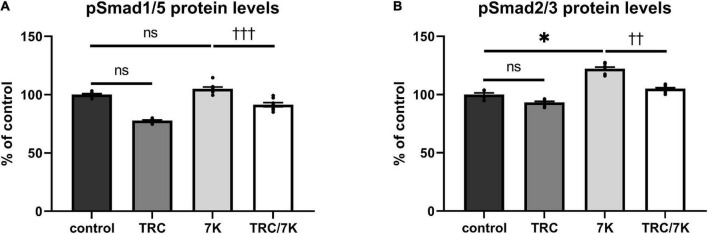
Effect of TRC105 on 7K-induced Eng signaling pathway in HAoECs. **(A)** pSmad1/5 protein levels; **(B)** pSmad2/3 protein levels. To analyze Smad signaling, cells were serum-starved in EBM-2 medium for 22 h and then pretreated with 300 μg/ml TRC105 for 90 min. HAoECs were stimulated with 10 μg/ml 7K for 30 min after TRC105 pretreatment. The data are presented as mean ± S.E.M (*n* = 6). ns, *p* ≥ 0.05; *p < 0.05, using Kruskal–Wallis test for comparisons among control, TRC105, and 7K, and ^††^*p* < 0.01; ^†††^*p* < 0.001, using Mann–Whitney test for comparison between 7K *vs* TRC/7K.

To explore the effects of TRC105 on the Eng signaling pathway, serum-starved HAoECs were exposed to TRC105 with or without 7K treatment. As shown in Figures 3A,B, treatment with 7K resulted in significant induction of pSmad2/3 protein levels but not pSmad1/5 protein levels, which is in line with our previous publication (33).

Interestingly, the addition of TRC105 to 7K-treated cells (TRC/7K) resulted in a decrease of pSmad1/5 and pSmad2/3 protein levels compared to 7K, suggesting that TRC105 inhibits 7K-induced Smad signaling.

### TRC105 induces protein levels of cell adhesion molecules

**FIGURE 4:**
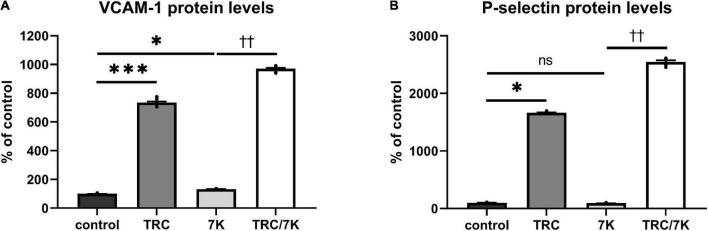
Effect of TRC105 on 7K-induced protein levels of cell adhesion molecules in HAoECs. **(A)** VCAM-1 protein levels; **(B)** P-selectin protein levels. For the detailed experimental design, please see Figure 1. The data are presented as mean ± S.E.M (*n* = 6). ns, *p* ≥ 0.05; *p < 0.05; ***p < 0.001, using Kruskal–Wallis test for comparisons among control, TRC and 7K, and ^††^*p* < 0.01, using Mann–Whitney test for comparison between 7K *vs*. TRC/7K.

To investigate the TRC105 effect on biomarkers of inflammation and endothelial dysfunction, we measured protein levels of the cell adhesion molecules VCAM-1 and P-selectin in HAoECs exposed to TRC105 with or without 7K treatment. We observed an increase in the protein levels of VCAM-1 and P-selectin in both groups exposed to TRC105, independently of 7K treatment (Figures 4A,B), suggesting the proinflammatory potential of TRC105 in endothelial cells.

### TRC105 prevents 7K-induced adhesion and transmigration of monocytes through HAoECs monolayer

**FIGURE 5:**
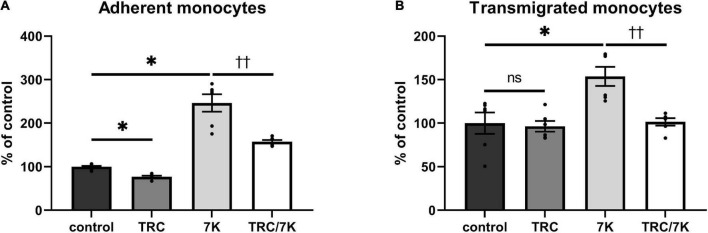
Effect of TRC105 on 7K-induced adhesion and transmigration of monocytes through monolayers of HAoECs. **(A)** adherent monocytes; **(B)** transmigrated monocytes. For the detailed experimental design, please see Figure 1. The data are presented as mean ± S.E.M (*n* = 6). ns, *p* ≥ 0.05; *p < 0.05, using Kruskal–Wallis test for comparisons among control, TRC and 7K, and ^††^*p* < 0.01, using Mann–Whitney test for comparison between 7K *vs* TRC/7K.

Endothelium’s functional properties were evaluated using 7K-induced adhesion and transmigration of monocytes through HAoECs monolayers, as previously described (33). As shown in Figure 5A, TRC105 was able to prevent the 7K-induced adhesion of THP-1 monocytes to the endothelial monolayer. Similarly, TRC105 also prevented THP-1 monocytes transmigration through the monolayer of HAoECs (Figure 5B), suggesting that TRC105 ameliorates 7K-induced endothelial dysfunction in HAoECs.

### Hyperglycemia induces Eng mRNA and protein expression levels

**FIGURE 6:**
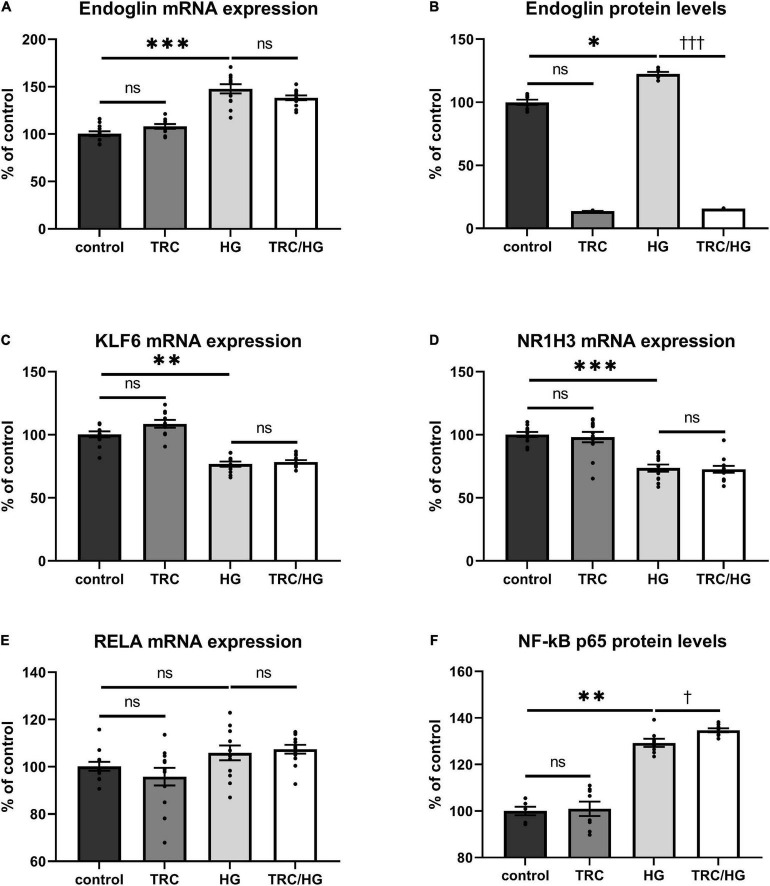
Effect of TRC105 on hyperglycemia-induced mRNA and protein expression of Eng, and on the Eng transcription factors KLF6, NR1H3, and RELA in HAoECs. **(A)** Eng mRNA expression. **(B)** Eng protein levels. **(C)** KLF6 mRNA expression. **(D)** NR1H3 mRNA expression. **(E)** RELA mRNA expression. **(F)** NF-κB p65 protein levels. For the detailed experimental design, please see Figure 1. The data are presented as mean ± S.E.M (*n* = 6). ns, *p* ≥ 0.05; *p < 0.05; **p < 0.01; ***p < 0.001, using Kruskal–Wallis test for comparisons between control, TRC, and HG, and ^†^*p* < 0.05; ^†††^*p* < 0.001, using Mann–Whitney test for comparison between HG *vs*. TRC/HG.

Exposure of HAoECs to hyperglycemia resulted in significantly increased Eng mRNA expression (Figure 6A) and protein (Figure 6B) levels. Surprisingly, mRNA expression of both Eng transcription factors (*KLF6* and *NR1H3)* was significantly decreased in the HG group (Figures 6C,D). Despite no significant change being detected in mRNA expression of *RELA* (Figure 6E), protein expression of NF-κB p65 was significantly increased (Figure 6F), similarly to after 7K treatment.

To determine the impact of TRC105 on hyperglycemia-induced Eng expression, we measured mRNA expression and protein levels of Eng and its transcription factors. Eng protein levels were significantly decreased in TRC/HG group compared to HG group (Figure 6B). However, no significant change in *Eng* mRNA expression was found after exposure to TRC105 (TRC/HG) when compared to HG (Figure 6A). Similarly, there was no significant change in mRNA expression of Eng transcription factors *KLF6*, *NR1H3*, and *RELA* (Figures 6C–E) between HG and TRC/HG. Nevertheless, we observed a slight increase in NF-κB p65 protein levels after administration of TRC105 (TRC/HG) when compared to HG (Figure 6F).

Together, these data suggest that TRC105 blocks hyperglycemia-induced Eng expression in a similar manner as in hypercholesterolemic conditions (7K treatment).

### Hyperglycemia induces pSmad1/5 but not pSmad2/3 signaling pathway

**FIGURE 7:**
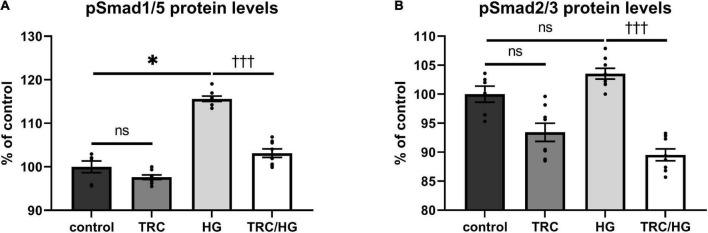
Effect of TRC105 on hyperglycemia-induced Eng signaling pathway in HAoECs. **(A)** pSmad1/5 protein levels; **(B)** pSmad2/3 protein levels. For Smad signaling analysis, cells were serum-starved in EBM-2 medium for 22 h and then pretreated with 300 μg/ml TRC105 for 90 min. Then, cells were stimulated with 45 mM HG for 30 min after TRC105 pretreatment. Data are presented as mean ± S.E.M (*n* = 6). ns, *p* ≥ 0.05; *p < 0.05, using Kruskal–Wallis test for comparisons among control, TRC and HG, and ^†††^*p* < 0.001, using Mann–Whitney test for comparison between HG *vs*. TRC/HG.

To evaluate the effects of hyperglycemia on Eng signaling cascade, serum-starved cells were premedicated with HG. This treatment resulted in mild but significant induction of pSmad1/5 (Figure 7A), but not pSmad2/3 (Figure 7B) protein levels compared to untreated control.

TRC105 treatment of HG cells (TRC/HG) decreased both pSmad1/5 (Figure 7A) and pSmad2/3 (Figure 7B) protein levels compared to cells treated only with HG. These data suggest that TRC105 treatment inhibits HG-induced pSmad expression in a similar manner as observed with 7K-induced pSmad expression.

### Hyperglycemia and TRC105 induce expression of cell adhesion molecules

**FIGURE 8:**
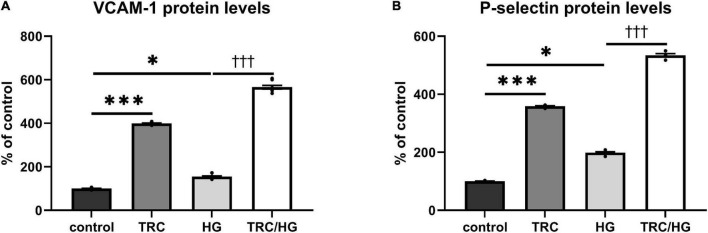
Effect of TRC105 on hyperglycemia-induced protein levels of cell adhesion molecules in HAoECs. **(A)** VCAM-1 protein levels. **(B)** P-selectin protein levels. For the detailed experimental design, please see Figure 1. The data are presented as mean ± S.E.M (*n* = 6). ns, *p* ≥ 0.05; *p < 0.05; ***p < 0.001, using Kruskal–Wallis test for comparisons among control, TRC and HG, and ^†††^*p* < 0.001, using Mann–Whitney test for comparison between HG *vs*. TRC/HG.

In line with previous studies (45, 46), the treatment of endothelial cells with HG resulted in an increase of cell adhesion molecules VCAM-1 (Figure 8A) and P-selectin (Figure 8B). In addition, exposure to TRC105 led to an even further significant increase in VCAM-1 and P-selectin protein levels in both control and HG-treated cells, suggesting the proinflammatory potential of TRC105 on endothelial cells. This proinflammatory effect of TRC105 was similar to that observed under hypercholesterolemic conditions (7K-treated cells).

### TRC105 prevents hyperglycemia-induced adhesion and transmigration of monocytes through HAoECs monolayer

**FIGURE 9:**
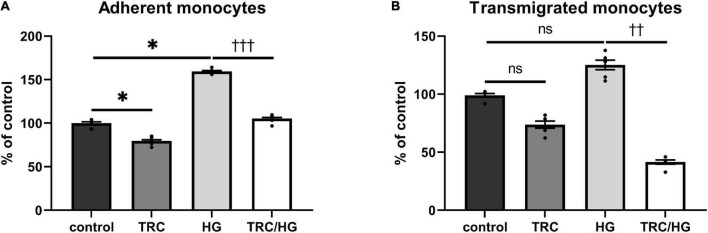
Effect of TRC105 on hyperglycemia-induced adhesion and transmigration of monocytes through HAoECs monolayer. **(A)** adherent monocytes; **(B)** transmigrated monocytes. For the detailed experimental design, please see Figure 1. Data are presented as mean ± S.E.M (*n* = 6). ns, *p* ≥ 0.05; *p < 0.05, using Kruskal–Wallis test for comparisons among control, TRC, and HG, and ^††^*p* < 0.01; ^†††^*p* < 0.001, using Mann–Whitney test for comparison between HG *vs*. TRC/HG.

The functional status of the endothelium was assessed by adhesion and transmigration assays. Exposure of cells to high glucose significantly increased the adhesion of monocytes to HAoECs (Figure 9A). We observed a similar trend in transmigration assays; however, this was not significant (Figure 9B).

Similarly, to 7K-induced endothelial dysfunction, HG-treated cells exposed to TRC105 (TRC/HG) demonstrated a significant decrease in adhesion (Figure 9A) and transmigration (Figure 9B) of monocytes via endothelial monolayer when compared to HG group. These results suggest that TRC105 prevents cell adhesion and transmigration associated with hyperglycemia-induced endothelial dysfunction.

## Discussion

The results of this study show for the first time that direct blockage of Eng and its downstream signaling can potentially prevent hypercholesterolemia- or hyperglycemia-induced development of endothelial dysfunction.

Previous studies emphasized the important role of Eng in inflammation-induced endothelial dysfunction and transendothelial migration (32). Similarly, Eng expression was increased in hypercholesterolemia-induced adhesion and transmigration of monocytes via endothelium (33).

Hyperglycemia represents a risk factor for the development of endothelial dysfunction in both experimental and clinical studies (47, 48). La Sala et al. showed an increase in Eng expression in HUVECs after glucose treatment (35). Hence, both risk factors (hypercholesterolemia and hyperglycemia) related to the development of endothelial dysfunction increase Eng mRNA expression and protein levels in endothelial cells.

TRC105 (carotuximab) is an anti-endoglin monoclonal antibody that was initially developed for use in oncology, and it is currently in clinical trials for acute macular degeneration (39). In this study, TRC105 was used as a model of pharmacological substance that is able to directly modulate Eng and its signaling during the development of endothelial dysfunction.

The first aim of this study was to reveal the potential differences between hypercholesterolemia- and hyperglycemia-induced Eng expression, signaling, and function (adhesion and transmigration of monocytes) in the endothelium. It was recently demonstrated that 7K-induced Eng expression and protein levels in HAoECs is regulated by the simultaneous interplay among three transcription factors – KLF6, NR1H3 (which encodes LXR protein) and RELA (encodes NF-κB p65 protein) (33). To the best of our knowledge, there is only one paper studying HG-mediated expression of Eng transcription factors. The authors reported that high glucose treatment increased the Eng mRNA expression and protein levels, together with KLF6 and HIF-1α (which are regulated by NF-κB) (35). Similarly, HG treatment increased mRNA and protein expression of Eng in HAoECs in this study. Nevertheless, we did not observe the same regulation of Eng expression by the transcription factors mentioned above. Under hyperglycemic conditions, we found that the mRNA expression of KLF6 and NR1H3 was significantly decreased. Despite the fact that the mRNA expression of RELA was not changed, NF-κB p65 protein levels were significantly increased in a similar manner as in 7K treatment, suggesting that NF-κB might be essential in both 7K- and HG-induced expression of Eng, and thus NF-κB activation is crucial for Eng expression, as demonstrated previously (49, 50).

Despite different regulation of Eng expression by its transcription factors, functional analysis showed that both 7K and HG increased protein levels of VCAM-1, adhesion, and transmigration of monocytes via endothelium compared to non-treated cells. This confirms the development of endothelial dysfunction in this experimental design, which was the crucial condition for the execution of the second aim of this study.

The second aim of this study was to evaluate the hypothesis that direct blockage of Eng prevents the development of 7K- and HG-induced endothelial dysfunction in order to demonstrate the importance of pharmacological modulation of Eng with respect to the development of endothelial dysfunction.

TRC105 did not significantly affect the mRNA expression of Eng in control 7K- and HG-stimulated cells, which is in agreement with the previously published study (51). Similarly, there was no change in the mRNA expression of Eng transcription factors KLF6, NR1H3, and RELA. Other transcription factors have been reported to modulate Eng gene expression, including Sp1 and HIF-1α, which regulate basal (43, 44) or hypoxia-induced (42) Eng transcription, respectively. However, given the lack of TRC105 effect on Eng mRNA levels, they were not investigated in the current study. However, Eng protein levels were significantly decreased, suggesting blockage of Eng by TRC105. Thus, we aimed to reveal the impact of this blockage on Eng related signaling.

Eng was demonstrated to affect Smad signaling either by activating ALK-1/pSmad1/5 signaling cascade, which leads to stimulation of migration, proliferation, and angiogenesis, or ALK-5/pSmad2/3 signaling pathway, which promotes cell senescence and suppresses angiogenesis (29, 31). Moreover, Kumar et al. observed that TRC105 abrogated TGF-β-induced Smad1/5/8 activation, whereas its effect on Smad2/3 was enhanced (52). Nevertheless, Liu et al. demonstrated that Smad2/3 phosphorylation was inhibited after TRC105, even though to a lesser extent than the Smad1/5/8 phosphorylation (41). This study showed that TRC105 treatment suppressed 7K- and HG-mediated increases of both pSmad1/5 and pSmad2/3 protein levels, suggesting inhibition of Eng signaling. Therefore, we propose that TRC105 blocks Eng on the cell surface, which leads to decreased intracellular signaling of Eng in HAoECs. It is of interest to mention that individual Smads (e.g., Smad2 and Smad3) have distinct roles with respect to regulation of different genes. However, in the current study, we did not explore which of the individual Smads was affected by TRC105 treatment, which might open a new area for the future research.

Cell adhesion molecules, including VCAM-1 and P-selectin, which are expressed on the vascular endothelium and circulating leukocytes in response to various inflammatory stimuli, are the primary mediators of the recruitment of inflammatory cells from the circulation to vessel intima (53). TRC105, combined with 7K or HG, resulted in a significant increase of P-selectin and VCAM-1 expression, suggesting that TRC105 promotes 7K- and HG-induced inflammation. However, in spite of the increased levels of P-selectin and VCAM-1, the blockage of Eng and its signaling by a pharmacological approach (TRC105) in this study prevented 7K- and HG-induced adhesion and transmigration via endothelium. These results suggest that Eng blockage prevails over the adhesive properties of VCAM-1 and P-selectin, further supporting the crucial role of Eng in preventing 7K- and HG-induced endothelial dysfunction. This conclusion is in agreement with a previous report from our research group (33). We found that stimulation of cells with 7K led to increased levels of endoglin and other adhesion molecules, as well as increased adhesion and transmigration of monocytes through endothelial monolayer. However, when endoglin levels were reduced with specific siRNA, a decreased adhesion and transmigration of monocytes through endothelial cells was observed, suggesting that in this experimental setting endoglin plays a crucial role of in the adhesion/transmigration process (33). The molecular mechanism by which endoglin is involved in adhesion/transmigration could be related to the fact that the extracellular domain of Eng has been proved to play an essential role in integrin-mediated cell adhesion (54). It was demonstrated that Eng interacts with leukocyte integrin α5β1 via its RGD motif *in vitro*, and correspondingly, inflammation-induced leukocyte transendothelial migration was significantly lower in *Eng*^±^ mice than *Eng*^+/+^ mice *in vivo*, suggesting a regulatory role for Eng in transendothelial leukocyte trafficking (32).

**FIGURE 10:**
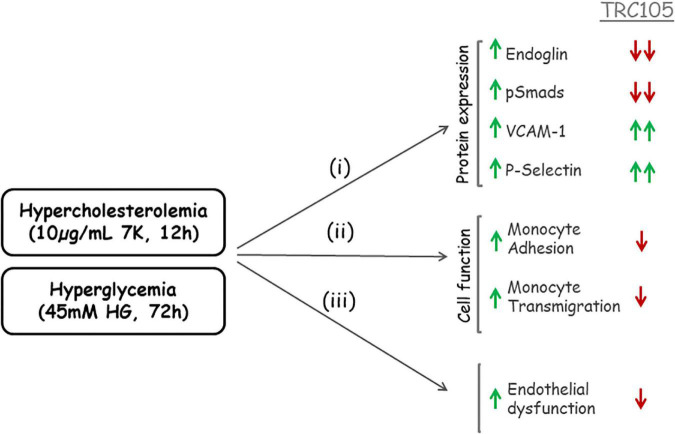
Schematic representation of TRC105 effects on human aortic endothelial cells under hypercholesterolemic (treated with 10 μg/ml 7-ketocholesterol [7K] for 12 h) or hyperglycemic (treated with 45 mM high glucose [HG] for 72 h) conditions. Hypercholesterolemia or hyperglycemia stimulates (i) protein expression of Eng, pSmads, vascular cell adhesion molecule 1 (VCAM-1), and P-selectin in endothelial cells; and (ii) monocyte adhesion to endothelial cells and monocyte transmigration through endothelial cell monolayers. The overall effect of hypercholesterolemia or hyperglycemia is endothelial dysfunction (iii). TRC105 inhibits 7K- and HG-induced expression of Eng and pSmads, while enhancing even further expression of VCAM-1 and P-selectin. TRC105 also inhibits 7K- and HG-induced adhesion of monocytes to endothelial cells and transmigration of monocytes through the endothelium, regardless of increased expression of cell adhesion molecules.

In summary, a schematic representation of TRC105 effects on 7K- and HG-induced expression of Eng is summarized in Figure 10.

TRC105, along with several other anti-endoglin antibodies, have been shown to induce the shedding of sEng (52). Therefore, it is likely that this mechanism of sEng release may contribute to the Eng downregulation observed in this work. In this regard, one limitation of this study is that levels of sEng could not be measured since TRC105 interferes with the ELISA method (R&D Quantikine CD105 Immunoassay kit) as described by Liu et al. (51).

**FIGURE 11:**
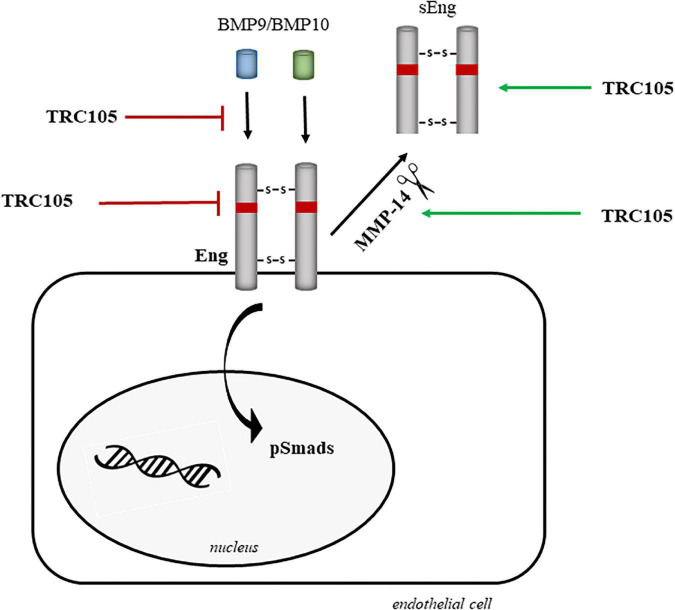
Hypothetical model of the molecular mechanisms underlying the effects of TRC105 by targeting Eng in human aortic endothelial cells under hypercholesterolemic (treated with 7-ketocholesterol [7K]) or hyperglycemic (treated with high glucose [HG]) conditions. TRC105 can inhibit: (i) the binding of the Eng ligands BMP9 and BMP10 (40); (ii) the expression of membrane-bound Eng; and (iii) the phosphorylation of Smads, which are downstream targets of Eng. TRC105 can also stimulate the protein expression of the metalloproteinase 14 (MMP-14), which in turn cleaves membrane-bound Eng and releases sEng (52).

Figure 11 summarizes the potential mechanisms by which TRC105 could target and downregulate Eng biological activity, including inhibition of (i) BMP9/BMP10 binding, (ii) Eng expression, and (iii) phosphorylation of Smads due to blockage of Eng. These inhibitory activities can be reinforced by the TRC105-dependent increased expression of MMP14, which in turn would target membrane-bound Eng, followed by the release of sEng, and therefore decreasing the levels of membrane-bound Eng. Further investigation will be needed to elucidate the exact molecular mechanism by which TRC105 modulates endothelial dysfunction.

In conclusion, anti-endoglin monoclonal antibody TRC105 prevents 7-ketocholesterol- and high glucose-induced adhesion and transmigration of monocytes through endothelial monolayer by blocking Eng and its signaling, regardless of increased expression of cell adhesion molecules. This suggests a critical role of Eng in the development of endothelial dysfunction under hypercholesterolemic and hyperglycemic conditions, which makes Eng an interesting pharmacological target in disorders related to elevated cholesterol and glucose levels. However, future studies in preclinical animal models are the first necessary step to confirm these *in vitro* data and suggestions.

## Data availability statement

The original contributions presented in this study are included in the article, further inquiries can be directed to the corresponding author.

## Author contributions

KT and PN: conceptualization, validation, data curation, and writing − original draft preparation. KT, MVi, and MVa: methodology. KT, CB, and PN: formal analysis. II, MVi, RH, SE, ZS, BV, CT, and CB: writing − review and editing. KT and CB: visualization. PN: supervision, project administration, and funding acquisition. All authors read and agreed to the published version of the manuscript.
